# Vergence and Accommodative Dysfunctions in Emmetropic and Myopic Chinese Young Adults

**DOI:** 10.1155/2019/5904903

**Published:** 2019-07-17

**Authors:** Martin Ming-Leung Ma, Anna Chwee Hong Yeo, Mitchell Scheiman, Xiang Chen

**Affiliations:** ^1^State Key Laboratory of Ophthalmology, Zhongshan Ophthalmic Center, Sun Yat-sen University, Guangzhou 510060, China; ^2^Research & Development Asia, Essilor International, Kallang Bahru, Singapore; ^3^Pennsylvania College of Optometry at Salus University, Philadelphia, PA, USA

## Abstract

**Purpose:**

To investigate the association between refractive error and common binocular vision and accommodative dysfunctions in Chinese adults and to report the percentage of these disorders in this sample population.

**Methods:**

This was a single-site, prospective cross-sectional clinic-based study. A total of 415 Chinese participants aged between 21 and 38 years were grouped into 4 refractive error groups (emmetropia, low, moderate, and high myopia) based on the spherical equivalent power of noncycloplegic refraction. Baseline testing including binocular vision and accommodative testing was performed on all eligible participants. A multiple-sign classification system was used to analyze these data for the diagnosis of common nonstrabismic binocular vision and accommodative dysfunctions. Associations between the diagnosis and refractive error groupings were examined by the chi-square test for the linear trend.

**Results:**

Associations with refractive error groupings were found for convergence insufficiency (*p*=0.008, *r* = −0.13) and divergence insufficiency (*p*=0.008, *r* = 0.131). The 3 most common dysfunctions in this sample population were basic exophoria (10.8%), convergence insufficiency (9.6%), and divergence insufficiency (7.0%). Approximately 40% of the sample population demonstrated at least one type of binocular vision dysfunction.

**Conclusion:**

Convergence insufficiency and divergence insufficiency were associated with refractive error groupings. Binocular vision dysfunction was a common finding in this sample population.

## 1. Introduction

Binocular vision dysfunction is reported to be the second most common optometric condition other than refractive error in the pediatric population [1]. Binocular vision dysfunction can cause a wide variety of symptoms including but not limited to blurriness, diplopia, headache, motion sickness, and poor concentration [2]. Most studies are performed in pediatric populations [3–5] or involve high school/university students [6–9]; however, little [10, 11] is known about the prevalence of these anomalies in young adults. This population is of great interest because college students and the young adult workforce have perhaps the greatest amount of close work of any population, and the presence of binocular and accommodative dysfunction may result in visual symptoms that affect work and leisure activities. To our knowledge, prevalence estimates for binocular and accommodative dysfunction in the Chinese adult population have not been reported. The lack of these data could have a considerable impact by preventing disease burden quantification and evidence-based healthcare planning. Furthermore, if binocular vision and accommodative dysfunctions are found to be prevalent in the Chinese population, more research should be performed in this area.

While one previous report suggested no correlation between refractive error and vergence dysfunction [12], our data from a previous report [13] demonstrated an association between refractive error grouping and the prevalence of convergence insufficiency in a sample of Chinese teenagers [13]. In this study, we test the hypothesis that there may also be associations between refractive error grouping and the distribution of vergence and accommodative dysfunctions in young Chinese adults. Such associations are particularly important since myopia is a prevalent condition in China [14]. If these dysfunctions are found to be more prevalent with myopia, special attention will be necessary when examining Chinese patients with myopia.

The main objective of this paper is to determine whether refractive error grouping is associated with the distribution of different vergence and accommodative dysfunctions in the Chinese adult population. Secondly, we will also report the percentages of common nonstrabismic vergence and accommodative anomalies in this population.

## 2. Method

This was a single-site, prospective cross-sectional clinic-based study. Participants were recruited through advertisement. All procedures met the tenets of the Declaration of Helsinki and were approved by the ethics committee of Zhongshan Ophthalmic Centre. Written consent was obtained from participants before any study procedures were performed. All examinations took place at Zhongshan Ophthalmic Centre between November 2014 and March 2015.

### 2.1. Participants

The inclusion criteria were as follows: (1) volunteer subject who was willing to follow the protocol and able to read, comprehend, and sign the informed consent form; (2) age between 21 and 38 years; (3) unremarkable general and ocular health; and (4) best-corrected visual acuity of at least 6/7.5 in each eye. Subjects with strabismus, diabetes, active ocular, and neurologic or muscular diseases, or a history of refractive surgery were excluded. Based on noncycloplegic refraction, subjects with more than +0.75 D hyperopia (spherical equivalent), 2 D astigmatism, or 1.5 D anisometropia were also excluded.

The enrolled participants were grouped into 4 refractive error groups based on the spherical equivalent power of noncycloplegic refraction. The criteria of these 4 groups were as follows: (1) emmetropia, from +0.75 to −0.75; (2) low myopia, from >−0.75 to −3.00; (3) moderate myopia, from >−3.00 to −6.00; and (4) high myopia, >−6.00. If two eyes were grouped differently, the participant was grouped based on the eye with the lower spherical equivalent power. The number of participants was designed arbitrarily to be 107 in each group.

### 2.2. Clinical Test Procedures

A preliminary examination (demographic data, habitual visual acuity at distance and near, lensometry of habitual glasses, cover test at distance and near to determine whether strabismus was present, and noncycloplegic objective and subjective refraction) was performed on all participants by trained optometrists. A complete binocular vision and accommodative evaluation was then performed with full subjective refraction on all eligible participants.

A modified Thorington card was used to measure phoria at 40 cm but not at distance due to its limited range of distance measurement (4△ esodeviation to 4△ exodeviation). The phoria at distance was measured using the Maddox rod technique with a phoropter. These two phoria measurements were used to define vergence dysfunction. Positive and negative fusional vergences were measured using a 20/30 letter target and Risley rotating prism on phoropter at 40 cm and 6 m based on subjective response from patients. The average of 3 measurements was used for analysis. The near point of convergence was measured with the Royal Air Force rule. The average of 3 break point measurements was used for analysis. The near point of convergence break point was either subjectively indicated when the patient report diplopia or objectively indicated when the patient demonstrated suppression during testing.

The monocular accommodative amplitude (right eye only) was evaluated three times. The patient was instructed to report the “first sustained blur” as the target was moved slowly (2 cm/second) towards the eye. Monocular accommodative facility was measured (right eye only) with ±1.50 D and ±1.00 D flippers for participants aged from 21 to 29 years and from 30 to 38 years, respectively [15]. Vergence facility was evaluated using a prism flipper with 3Δ Base-in/12Δ Base-out at 40 cm. Participants were instructed to report clarity (say “clear”) as soon as the letters were single and clear. The number of cycles per minute was recorded. A column of 20/30 letters was used as the fixation target for the accommodative amplitude test and facility tests. There was no specific requirement for the sequence or the waiting time between repeated measurements or between different tests.

### 2.3. Diagnostic Criteria

The nonstrabismic vergence and accommodative dysfunctions reported in this study included convergence insufficiency and excess, basic exophoria and esophoria, divergence insufficiency and excess, fusional vergence dysfunction, accommodative insufficiency, and infacility. A multiple-sign classification system was used for diagnosis and is shown in Table 1. This system was based on criteria used in other studies [1, 3–11, 16, 17].

For vergence dysfunction, if the participant was diagnosed with more than one type of dysfunction, the investigator (M.M.) reviewed their case record to determine the most appropriate diagnosis individually using clinical judgment. For accommodative dysfunction, participants could be diagnosed with both accommodative infacility and insufficiency. Participants could also be diagnosed with both vergence and accommodative dysfunctions. Normal binocular vision was a diagnosis of exclusion and was diagnosed only if a participant did not meet any of the diagnostic classifications listed in Table 1.

### 2.4. Statistical Analysis

Associations between the dysfunction diagnosis and refractive error groupings were examined using the chi-square test for the linear trend. For dysfunctions that showed an association with refractive error grouping, logistic regression was performed to compare the prevalence of dysfunctions in different refractive error grouping against that in emmetropia group. One-way ANOVA was used to compare the baseline characteristics. The significance level was set at 0.05 for all tests. Due to the abundance of data, detailed analysis of binocular vision and accommodation test data will be presented in a separate report.

## 3. Results

Of the 422 screened potential participants, 415 participants were eligible and participated in the study. The major reason for exclusion was the presence of strabismus. The baseline characteristics are shown in Table 2. The low myopia and moderate myopia groups each comprised 107 participants, while the emmetropia and high-myopia groups only comprised of 96 and 106 participants, respectively. The number of participants in the emmetropia and high-myopia groups did not reach the intended target (i.e., 107 participant) because we were not able to recruit sufficient participants within the planned recruitment period.

The one-way ANOVA indicated that there were significant differences in age (dF = 3, *p* < 0.001), noncycloplegic objective (dF = 3, *p* < 0.001), and subjective refraction (dF = 3, *p* < 0.001). Regarding age, Tukey's honestly significant difference post hoc test showed that only the differences between emmetropes and moderate myopes (95% CI = −4.2 to −1.1, *p* < 0.001), emmetropes and high myopes (95% CI = −3.2 to −0.02, *p* < 0.001), and low myopes and moderate myopes (95% CI = −3.6 to −0.5, *p*=0.0025) were significant.

The distribution of the different binocular vision and accommodative dysfunctions is summarized in Table 3. The sum of all classifications was not equal to 100% because participants could be diagnosed with more than one dysfunction. For example, participants could be diagnosed with convergence insufficiency, accommodative insufficiency, and infacility. The mean values of various binocular vision and accommodation test results are presented in Table 4.

Regarding the total sample, the 3 most common dysfunctions were basic exophoria, convergence insufficiency, and divergence insufficiency. The most common dysfunctions for emmetropes and low, moderate, and high myopes were convergence insufficiency, basic exophoria, divergence insufficiency, and basic exophoria, respectively. Analysis indicated that only convergence insufficiency (*p*=0.008, *r* = −0.13) and divergence insufficiency (*p*=0.008, *r* = 0.131) were associated with refractive error groupings. Convergence insufficiency was more likely to occur in patients with a lower degree of myopia, while divergence insufficiency was more likely to occur in patients with a higher degree of myopia. Linear regression showed that the frequency of convergence insufficiency in moderate myopes (*p*=0.043) and high myopes (*p*=0.043) was lower than that of the emmetrope group, while the frequency of divergence insufficiency in high myopes (*p*=0.029) was higher than that of the emmetrope group. The diagnosis of normal binocular vision was not associated with refractive error groupings.

## 4. Discussion

The results of this study demonstrate that only convergence insufficiency and divergence insufficiency were associated with the established refractive error groupings, and binocular vision dysfunction was a common finding in this sample population.

Our data indicate that divergence insufficiency was more likely to occur in patients with a higher degree of myopia. Research on divergence insufficiency is limited, possibly due to its reported low prevalence [1, 3, 8, 11]. In a study by Kohmoto et al. [18], they stated that “we have frequently observed high-myopia patients with divergence insufficiency in the absence of other neurological disorders.” They suggested that high myopia with eyeball elongation was a risk factor for divergence insufficiency, and they proposed that divergence insufficiency was caused mechanically by nasal shifting of the superior rectus and inferior shifting of the lateral rectus, which were found in their divergence insufficiency patients [18]. Notably, however, the divergence insufficiency subjects in their study had more severe dysfunction than that of the participants in the current study. For example, diplopia at distance was present in all of their participants. In addition, we believe there might be other reasons for the etiology of this association. Otherwise, there should also be an association between refractive error grouping and other esodeviation-related conditions (i.e., basic esophoria).

Regarding convergence insufficiency, we previously demonstrated that this diagnosis is associated with refractive error in a clinic-based study involving Chinese teenagers [13]. In that study, the frequency of convergence insufficiency was found to be the highest in hyperopes (12.9%), followed by emmetropes (4%) and myopes (1.6%). A similar trend was also observed in this study. In this study, the frequency of convergence insufficiency was the highest in emmetropes (15.8%), followed by low myopes (12.1%), high myopes (6.6%), and moderate myopes (4.7%). Data from these two studies support the association between refractive error and convergence insufficiency. A previous study suggested no association between refractive error and convergence insufficiency [12]; however, the composition of the study population was quite different. In the study by Wajuihian [12], the sample size was 1056 participants, but there were only 61 low myopes and 2 moderate myopes. This small myopia sample size may have affected the results of the study, and the relationship between refractive error and convergence insufficiency would be difficult to detect. A possible explanation for the association between convergence insufficiency and refractive error grouping could be the base-in prismatic effect induced by myopic correction at near vision. This effect would decrease the convergence demand and could effectively increase the near positive fusional vergence range and the near point of convergence. Patients with a higher degree of myopia, therefore, may exhibit a higher level of near positive fusional vergence and closer near point of convergence and would be less likely to be classified as having convergence insufficiency than those with a lower degree of myopia. Another possible explanation is related to accommodation. When viewing a near object with distance spectacles correction, a corrected myope uses less accommodation than an emmetrope, whereas a corrected hypermetrope uses more accommodation than an emmetrope [19]. The effect of this factor on these associations is difficult to predict because of the cross-coupling between accommodation and vergence systems.

A key feature of this study is that we deliberately did not consider subjective symptoms when classifying participants with a diagnostic condition for two reasons. First, it is possible to meet all of the criteria for a diagnostic condition and be asymptomatic because of suppression or avoidance of visual activities that provoke symptoms [20]. Second, there is no validated questionnaire available to quantify the symptoms for all binocular vision and accommodative disorders. While the Convergence Insufficiency Symptom Survey has been validated to assess changes in symptoms after treatment, it has only been validated for use with convergence insufficiency.

The 3 most common binocular vision disorders in this study were basic exophoria, convergence insufficiency, and divergence insufficiency. Basic exophoria was seldom the most common diagnosis in prevalence studies [1, 3, 4, 8, 9, 11], with reported prevalence rates of 0.3% to 5.1% [1, 4, 6, 9–11]. It is likely that the discrepancy is related to the definition used in this study, which is more liberal than those used in previous studies. Previous authors have noted the difficulty of comparing prevalence data across studies [24]. For example, in a recent population-based study by Hussaindeen et al. [3], basic exophoria was defined as symptomatic participants having “equal amount of exophoria at distance and near” and at least 2 of the following signs: receded near point of the convergence break point, reduced positive fusional vergence, and binocular accommodative facility. They did not find a single patient with basic exophoria in a sample of 920 children. Participants who were diagnosed with basic exophoria in the current study might have been classified as having convergence insufficiency or divergence excess in the study by Hussaindeen et al. due to their strict requirement for phoria.

Convergence insufficiency is often found to be the most or second most common dysfunction in prevalence studies [1, 3, 4, 7–9], and this was also true in our study. In previous studies, the prevalence of convergence insufficiency ranged from 2.25% to 33% [3, 6, 7, 16, 17, 21, 22]. An interesting observation is that the frequency of convergence insufficiency found in this study (9.6%) is significantly higher than that in our previous school-based study [13] (2.7%). The testing methodology and diagnostic criteria used in these two studies were the same. However, the arbitrarily similar numbers of participants in the 4 refractive error groups may have skewed the results, as an association existed between refractive error and convergence insufficiency. It was an interesting finding that divergence insufficiency was the third most common dysfunction because it is usually found to be a relatively uncommon dysfunction [1, 3, 8, 11].

A number of studies have reported that convergence excess is the most or second most prevalent binocular vision dysfunction [1, 4, 7–9], although one study showed that it is the least prevalent condition [11]. The main reason for this discrepancy is believed to be the difference in diagnostic criteria used in the various studies. Finally, only 59.8% of the study population was classified as “normal binocular vision” and fell within the previously reported range of normal binocular vision, which ranged from 43.7% to 87.3% [3, 7–11]. This result indicated that binocular vision dysfunction was a common finding in this sample.

The strengths of our study include its prospective design and the use of a specified set of diagnostic criteria. The sample size in all 4 refractive error groups was relatively large. Specifically, there were enough numbers of moderate and high myopes, allowing us to meaningfully test for associations. Regarding limitations, it must be noted that this study is not a true prevalence study due to its clinic-based nature. Participants who were willing to participate may have had more ocular symptoms and might have provided an overestimation of binocular vision dysfunctions. Although only adult participants were included, refractive error was only determined by noncycloplegic refraction; therefore, the grouping of refractive error may potentially be different from that determined by cycloplegic refraction. More importantly, the number of participants in the different refractive error groups was set to be arbitrarily similar among the 4 groups. This does not reflect how these 4 groups of refractive error are distributed within the general population. For example, we found that it was more common to have divergence insufficiency in moderate and high myopes. In reality, if the proportion of moderate and high myopes is significantly less than that in our study, the true prevalence should be lower; the same applies to convergence insufficiency. In addition, test selection may had affected the result. For example, use of the Maddox rod technique during distant phoria measurement may induce proximal vergence, which may explain why participants were more esophoric at distance than at near, and were thus diagnosed with divergence insufficiency. Finally, it should be noted that most of the tests used to diagnose vergence and accommodative dysfunctions are subjective tests. There is a great need for objective measurements in this field. A recent example of objective measurement of fusional vergence was reported by Scheiman et al. [23].

Because there were differences in the percentage of binocular vision dysfunctions in different refractive error groupings, further analysis or studies on binocular vision function in different refractive error groupings should be performed, at least in Chinese adults. Further study on this area may help us to understand the etiology of the associations found in the current study. Additionally, standardization of diagnosis is a key but missing component in this field of study. Although there has recently been a trend toward adopting a multiple-sign classification system, the details differ greatly across studies. Efforts should be undertaken to unify evidence-based diagnostic criteria with standardized cutoff values, testing methodology including instrumentation, fixation targets, and even testing instruction.

## 5. Conclusion

In this study, we found that approximately 40% of the sample population demonstrated at least one type of binocular vision or accommodative dysfunction. Convergence insufficiency and divergence insufficiency were associated with refractive error grouping. The 3 most common dysfunctions in the sample population were basic exophoria, convergence insufficiency, and divergence insufficiency.

## Figures and Tables

**Table 1:** Diagnostic criteria for vergence and accommodative dysfunctions.

*Convergence Insufficiency*
*Requires 1, 2, and 3*
1. Near exophoria at least 4△ greater than distance exophoria
2. Near point of convergence break point ≥6 cm
3. Reduced near positive fusional vergence (break point ≤15△ or failed Sheard's criterion)

*Convergence Excess*
*Requires 1*
*Plus at least 1 finding from 2 to 3*
1. Near esophoria greater than distance esophoria by ≥4△
2. Reduced near negative fusional vergence (break point ≤7△ or failed Sheard's criterion)
3. Near vergence facility ≤12 cycle per minute

*Divergence Insufficiency*
*Requires 1 or 2* *+* *3*
1. Distance esophoria greater than near esophoria by ≥10△
2. Distance esophoria greater than near esophoria by ≥4△
3. Reduced distance negative fusional vergence (break point ≤4△ or failed Sheard's criterion)

*Divergence Excess*
*Requires 1 or 2* *+* *3*
1. Distance exophoria greater than near esophoria by ≥10△
2. Distance exophoria greater than near esophoria by ≥4△
3. Reduced distance positive fusional vergence (break point ≤11△ or failed Sheard's criterion)

*Basic Esophoria*
*Requires 1 and 2*
*Plus at least 1 finding from 3 to 4*
1. Difference between near and distance esophoria ≤3△
2. Participant needs to be esophoric at both distant and near
3. Reduced near or distance negative fusional vergence (near break point ≤7△ or distance break point ≤4△ or failed Sheard's criterion)
4. Near vergence facility ≤12 cycle per minute

*Basic Exophoria*
*Requires 1 and 2*
*Plus at least 1 finding from 3 to 5*
1. Difference between near and distance exophoria ≤3△
2. Participant needs to be exophoric at both distant and near
3. Reduced near or distance positive fusional vergence (near break point ≤15△ or distance break point ≤11△ or failed Sheard's criterion)
4. Near point of convergence break point ≥6 cm
5. Near vergence facility ≤12 cycle per minute

*Fusional Vergence Dysfunction*
*Requires 1 and 2*
*Plus at least 1 finding from 3 to 4*
1. No significant phoria at distance and near (distance: exophoria ≤2△ to orthophoria; near: exophoria ≤5△ to orthophoria)
2. No other vergence dysfunction diagnosed
3. Reduced near positive or negative fusional vergence (positive fusional vergence break point ≤15△ or negative fusional vergence break point (NFV) ≤7△ or failed Sheard's criterion)
4. Near vergence facility ≤12 cycle per minute

*Accommodative Insufficiency*
*Requires 1*
1. Monocular amplitude of accommodation ≥2 diopters below the minimum prediction (15-age/4)

*Accommodative Infacility*
*Requires 1*
1. Monocular accommodative infacility ≤6 cycle per minute

**Table 2:** Baseline characteristics.

	Emmetropes (*n*=95)	Low myopes (*n*=107)	Moderate myopes (*n*=107)	High myopes (*n*=106)	Total (*n*=415)	Comparison of 4 groups, *p* value
Male, *n* (%)	36 (38%)	29 (27%)	27 (25%)	37 (35%)	129 (31%)	
Age, mean ± SD	28.4 ± 4.7	27.8 ± 4.6	25.7 ± 3.9	26.8 ± 4.0	27.1 ± 4.4	<0.001
Noncycloplegic objective refraction, right eye spherical equivalent, D, mean ± SD	−0.54 ± 0.64	−2.16 ± 0.71	−4.73 ± 0.93	−8.26 ± 1.97	−4.01 ± 3.14	<0.001
Noncycloplegic subjective refraction, right eye spherical equivalent, D, mean ± SD	−0.47 ± 0.51	−2.10 ± 0.74	−4.72 ± 0.83	−8.35 ± 1.86	−4.00 ± 3.17	<0.001
Distance esophoria, △, mean ± SD	0.0 ± 3.2	0.0 ± 3.7	1.1 ± 4.8	0.0 ± 4.9	0.3 ± 4.3	0.19
Near esophoria, △, mean ± SD	−4.5 ± 5.3	−3.8 ± 6.0	−3.5 ± 6.2	−5.0 ± 6.7	−4.2 ± 6.1	0.26

**Table 3:** Distribution of accommodative and vergence dysfunction by refractive error groups.

	Emmetropes (*n*=95)		Low myopes (*n*=107)		Moderate myopes (*n*=107)		High myopes (*n*=106)		Total (*n*=415)		*p* value	Pearson's *r*
	*n*	%	*n*	%	*N*	%	*n*	%	*n*	%		
Convergence insufficiency	15	15.8	13	12.1	5	4.7	7	6.6	40	9.6	0.008	−0.130
Convergence excess	1	1.1	1	0.9	1	0.9	1	0.9	4	1.0	1.000	−0.004
Divergence insufficiency	4	4.2	2	1.9	11	10.3	12	11.3	29	7.0	0.008	0.131
Divergence excess	0	0.0	1	0.9	1	0.9	1	0.9	3	0.7	0.619	0.036
Basic esophoria	3	3.2	10	9.3	6	5.6	2	1.9	21	5.1	0.420	−0.043
Basic exophoria	8	8.4	16	15.0	6	5.6	15	14.2	45	10.8	0.668	0.024
Fusional vergence dysfunction	5	5.3	1	0.9	2	1.9	1	0.9	9	2.2	0.093	−0.088
Accommodative insufficiency	8	8.4	3	2.8	4	3.7	3	2.8	18	4.3	0.102	−0.083
Accommodative infacility	2	2.1	3	2.8	2	1.9	2	1.9	9	2.2	0.881	−0.130
Normal binocular vision	51	53.7	61	57.0	72	67.3	64	60.4	248	59.8	0.174	0.067

**Table 4:** Mean values of various binocular vision and accommodation test results by refractive error groups.

	Emmetropes (*n*=95)	Low myopes (*n*=107)	Moderate myopes (*n*=107)	High myopes (*n*=106)	Total (*n*=415)
Distance positive fusional vergence break point, △, mean ± SD	19.9 ± 6.1	18.7 ± 6.5	22.3 ± 6.3	21.2 ± 6.7	20.1 ± 6.5
Distance negative fusional vergence break point, △, mean ± SD	11.7 ± 4.2	12.7 ± 3.7	12.8 ± 4.6	12.6 ± 3.9	12.3 ± 4.0
Near positive fusional vergence break point, △, mean ± SD	25.8 ± 7.7	25.7 ± 7.1	27.6 ± 8.3	28.2 ± 8.4	26.3 ± 7.9
Near negative fusional vergence break point, △, mean ± SD	21.4 ± 5.2	21.1 ± 5.4	22.1 ± 5.3	24.0 ± 6.0	21.8 ± 5.6
Vergence facility, cycles per minute, mean ± SD	16.0 ± 4.3	15.4 ± 4.0	16.5 ± 3.9	15.4 ± 3.0	15.6 ± 3.9
Monocular accommodative amplitude (right eye only), dioptre, mean ± SD	9.5 ± 2.5	8.8 ± 2.2	9.6 ± 2.0	10.0 ± 2.2	9.1 ± 2.2
Monocular accommodative facility (right eye only), cycles per minute, mean ± SD	14.2 ± 3.7	13.9 ± 3.7	14.6 ± 3.9	14.6 ± 4.3	14.3 ± 4.0
Near point of convergence break point, cm, mean ± SD	6.2 ± 2.7	6.5 ± 3.7	5.9 ± 2.8	5.8 ± 3.4	6.3 ± 3.2

## Data Availability

The data used to support the findings of this study are included within the article.
